# Association of *HLA-G* 3’ Untranslated Region Polymorphisms with Systemic Lupus Erythematosus in a Japanese Population: A Case-Control Association Study

**DOI:** 10.1371/journal.pone.0158065

**Published:** 2016-06-22

**Authors:** Yuki Hachiya, Aya Kawasaki, Shomi Oka, Yuya Kondo, Satoshi Ito, Isao Matsumoto, Makio Kusaoi, Hirofumi Amano, Akiko Suda, Keigo Setoguchi, Tatsuo Nagai, Kota Shimada, Shoji Sugii, Akira Okamoto, Noriyuki Chiba, Eiichi Suematsu, Shigeru Ohno, Masao Katayama, Hajime Kono, Shunsei Hirohata, Yoshinari Takasaki, Hiroshi Hashimoto, Takayuki Sumida, Shouhei Nagaoka, Shigeto Tohma, Hiroshi Furukawa, Naoyuki Tsuchiya

**Affiliations:** 1 Molecular and Genetic Epidemiology Laboratory, Faculty of Medicine, University of Tsukuba, Tsukuba, Ibaraki, Japan; 2 Master’s Program in Medical Sciences, Graduate School of Comprehensive Human Sciences, University of Tsukuba, Tsukuba, Ibaraki, Japan; 3 Clinical Research Center for Allergy and Rheumatology, Sagamihara Hospital, National Hospital Organization, Sagamihara, Kanagawa, Japan; 4 Department of Internal Medicine (Rheumatology), Faculty of Medicine, University of Tsukuba, Tsukuba, Ibaraki, Japan; 5 Department of Rheumatology, Niigata Rheumatic Center, Shibata, Niigata, Japan; 6 Department of Internal Medicine and Rheumatology, Juntendo University School of Medicine, Tokyo, Japan; 7 Department of Rheumatology, Yokohama Minami Kyosai Hospital, Yokohama, Kanagawa, Japan; 8 Center for Rheumatic Diseases, Yokohama City University Medical Center, Yokohama, Kanagawa, Japan; 9 Allergy and Immunological Diseases, Tokyo Metropolitan Cancer and Infectious Diseases Center Komagome Hospital, Tokyo, Japan; 10 Department of Rheumatology and Infectious Diseases, Kitasato University School of Medicine, Sagamihara, Japan; 11 Department of Rheumatology, Tokyo Metropolitan Tama Medical Center, Fuchu, Tokyo, Japan; 12 Department of Rheumatology, Himeji Medical Center, National Hospital Organization, Himeji, Hyogo, Japan; 13 Department of Rheumatology, Morioka Hospital, National Hospital Organization, Morioka, Iwate, Japan; 14 Department of Internal Medicine and Rheumatology, Clinical Research Institute, Kyushu Medical Center, National Hospital Organization, Fukuoka, Fukuoka, Japan; 15 Department of Internal Medicine, Nagoya Medical Center, National Hospital Organization, Nagoya, Aichi, Japan; 16 Department of Internal Medicine, Teikyo University School of Medicine, Tokyo, Japan; 17 Juntendo University School of Medicine, Tokyo, Japan; Nippon Medical School Graduate School of Medicine, JAPAN

## Abstract

HLA-G plays a role in fetal-maternal tolerance as well as immunoregulation, and has been suggested to be involved in autoimmune diseases and cancers. *HLA-G* encodes two potentially functional polymorphisms in the 3’ untranslated region, 14bp insertion/deletion (14bp indel, rs371194629) and a single nucleotide polymorphism rs1063320, previously reported to affect HLA-G expression level or splicing isoform and to be associated with susceptibility to systemic lupus erythematosus (SLE). However, the results of SLE association studies are inconsistent, probably due to the small sample size of each study and lack of consideration of linkage disequilibrium (LD) with *HLA-class II* haplotypes in each population. In this study, we performed association studies of these polymorphisms on 843 patients with SLE and 778 healthy controls in a Japanese population, in many of whom *HLA-DRB1* alleles have been genotyped at the four-digit level. LD was detected between DRB1*13:*02*, protective against multiple autoimmune diseases in the Japanese, and the rs1063320 G (*D’* = 0.86, *r*^*2*^ = 0.02) and with 14bp del (*D’* = 0.62, *r*^*2*^ = 0.01), but not between SLE-susceptible DRB1*15:*01* and *HLA-G*. Although significant association with overall SLE was not detected, 14bp ins allele was significantly associated with SLE with the age of onset <20 years, when compared with healthy controls (P = 0.0067, P_FDR_ = 0.039, OR 1.44, additive model) or with SLE patients with the age of onset ≥20 (P = 0.033, P_FDR_ = 0.0495, OR 2.09, additive model). This association remained significant after conditioning on DRB1*13:*02* or DRB1*15:*01*. On the other hand, significant association was detected between rs1063320 C and anti-RNP antibody and anti-Sm antibody positive SLE, which was dependent on negative LD with DRB1*13:*02*. eQTL analysis showed reduced *HLA-G* mRNA level in 14bp ins/ins individuals. In conclusion, our observations showed that *HLA-G* 14bp ins allele represents a genetic contribution on early-onset SLE independent of *DRB1*.

## Introduction

Systemic lupus erythematosus (SLE) is a prototype of systemic autoimmune diseases, caused by a combination of multiple genetic and environmental factors. Epidemiological studies showed higher prevalence in African American, Hispanic, and Asian rather than Caucasian populations [1]. Moreover, a number of twin studies convincingly demonstrated the evidence for the genetic component in SLE [2]. To date, more than 70 susceptibility regions were identified by genome-wide association studies (GWAS) as well as candidate gene studies [3].

The strongest association is localized in the major histocompatibility complex (*MHC*) region at 6p21.3 [4, 5]. Association of HLA-DRB1*15:*01* and DRB1*03:*01* haplotypes has been established in Caucasian and East Asian populations [6–9]. However, due to the extensive and long-range linkage disequilibrium (LD) encompassing not only *HLA* genes but also potentially relevant non-*HLA* genes, the search for the causative allele is still underway.

HLA-G, encoded near the telomeric end of *MHC* region, is one of the non-classical class I molecules. Although the role for HLA-G in fetal-maternal tolerance in placenta is well recognized, HLA-G is also physiologically expressed in the thymus, pancreas, lung, erythroid, macrophage, and endothelial precursors. Expression of HLA-G is induced under conditions such as inflammation and viral infection, and plays a role in immunoregulation [10, 11].

HLA-G interacts with immune inhibitory receptors expressed on NK cells, T cells, monocytes, and dendritic cells (DCs), such as leukocyte immunoglobulin-like receptor (LILR) B1, LILRB2, and killer cell immunoglobulin-like receptor (KIR) 2DL4 [11]. Previous studies have shown that HLA-G inhibited activation of CD8+ T cells and NK cells, proliferation of CD4+ T cells, differentiation of antigen presenting cells and B cells, and induced regulatory T cells and IL-10 producing DCs [10].

Unlike classical HLA, *HLA-G* gene has limited polymorphisms in its coding region; however, seven splicing isoforms including those generating soluble HLA-G (sHLA-G) as well as polymorphisms in the regulatory regions are known [12]. Among the latter, 14bp insertion polymorphism (14bp indel) (rs371194629) and a single nucleotide polymorphism (SNP), rs1063320 (G>C) in 3’ untranslated region (UTR), have been suggested to have functional significance. The 14bp insertion allele (ins) has been shown to be associated with alternative splicing and to result in deletion of 92 bp in exon 5 from mature mRNA, which then leads to low levels of soluble HLA-G (sHLA-G) [13]. Although some evidence also suggested that 14bp ins allele generated more stable mRNA than 14bp deletion (del) allele [14], most studies demonstrated significantly lower sHLA-G levels in individuals with 14bp ins/ins genotype [15, 16]. The association of 14bp indel with susceptibility to various diseases has been reported [10]. With respect to SLE, significant increase of 14bp ins allele was observed in a study from Italy [17], and although other studies in South American [18–21] and Asian [22] populations did not reach statistical significance, many of them showed a trend toward increase in 14bp ins allele frequency and/or association with clinical subsets, and meta-analyses reported significant association with susceptibility to SLE [21, 23, 24].

On the other hand, the SNP rs1063320 G>C is located at microRNA (miRNA) (miR-148a, miR-148b, miR-152) binding site. It was demonstrated that the G allele of rs1063320 increases the affinity of interaction with these miRNA and results in the reduction of HLA-G expression [25]. With respect to disease associations, significant increase of G allele has been reported in asthma [25] and in SLE [18, 19].

Interestingly, although located ~2.8 Mb apart, linkage disequilibrium was reported between some combinations of *HLA-DRB1* and *HLA-G* alleles [12, 26, 27]. For example, DRB1*03:*01* haplotype strongly associated with risk of SLE in the Caucasian populations is in LD with *HLA-G* 14bp ins allele [28].

Thus, several studies with limited sample size suggested association of *HLA-G* 3’UTR polymorphisms with SLE. However, most of the studies did not consider LD with *HLA-class II* SLE risk or protective haplotypes. Only Fernando et al. has taken LD into account, and demonstrated an independent genetic contribution of *HLA-G* region in a Filipino population [6]. However, they did not address the relationship between the marker and *HLA-G* 3’UTR 14bp indel nor rs1063320.

Association study of *HLA-G* with SLE in a Japanese population has not been reported. In view of the unique genetic background of the Japanese population where the allele frequency of DRB1*03:*01* is exceptionally low (0.1%) even among the East Asian populations [29], it was of interest to examine the association between *HLA-G* 3’UTR polymorphisms and SLE. In the present study, we carried out a case-control association study to examine the association between *HLA-G* and SLE, among many of whom *DRB1* alleles were already genotyped at the “four-digit” level (high resolution) [8]. We detected LD between SLE-protective DRB1*13:*02* and the rs1063320 G and with 14bp del, but not between SLE-susceptible DRB1*15:*01*. *HLA-G* 14bp ins allele was increased in SLE, especially those with the age of onset <20 years. This association remained significant when conditioned on DRB1*15:*01* and DRB1*13:*02*. These results suggested an independent role of *HLA-G* 14bp ins in the development of SLE in the patients with early age of onset.

## Subjects and Methods

### Subjects

Genomic DNA from 843 Japanese SLE patients and 778 healthy controls recruited at the universities and rheumatology centers were examined. All patients were classified as SLE according to the American College of Rheumatology classification criteria for SLE [30]. Presence or absence of nephropathy was classified according the same criteria [30]. Early-onset was defined as an age of onset of earlier than 20 years of age. The clinical characteristics of the patients are shown in Table 1.

**Table 1:** Patients and healthy controls examined in this study.

Subjects	n	%*
all SLE	843	
age of onset <20	144	18.6%
nephropathy (+)	397	49.6%
anti-dsDNA Ab (+)	654	82.4%
anti-Sm Ab (+)	252	32.5%
anti-RNP Ab (+)	254	40.4%
HC	778	

*Proportion of the patients of each subcategory among those with available data. Ab: antibody, HC: healthy controls.

All healthy controls were unrelated Japanese, among whom 578 were recruited at the research institutes in the Kanto area, where most of the patients were also recruited. In addition, genomic DNA from 200 healthy controls were derived from the B cell lines deposited by Japan Biological Informatics Consortium and purchased from the Health Science Research Resources Bank.

### Ethics statement

This study was reviewed and approved by the Ethics Committees of the following institutes: University of Tsukuba Faculty of Medicine, Sagamihara Hospital, National Hospital Organization, Juntendo University School of Medicine, Yokohama Minami Kyosai Hospital, Yokohama City University Medical Center, Tokyo Metropolitan Cancer and Infectious Diseases Center Komagome Hospital, Kitasato University School of Medicine, Tokyo Metropolitan Tama Medical Center, Himeji Medical Center, National Hospital Organization, Morioka Hospital, National Hospital Organization, Kyushu Medical Center, National Hospital Organization, Nagoya Medical Center, National Hospital Organization, and Teikyo University School of Medicine.

Written informed consent was obtained from all participants, except for some individuals who donated the blood for the purpose of medical genetics study before 2001, prior to the enforcement of the Ethics Guidelines for Human Genome/Gene Analysis Research by the Japanese government. From such individuals, oral informed consent for the genetics study had been obtained. In accordance with the Japanese Ethical Guidelines for Human Genome/Gene Analysis Research, such samples were anonymized in an unlinkable fashion, and were included in this study, after review and approval by the Ethics Committee of University of Tsukuba. This study was conducted according to the principles expressed in the Declaration of Helsinki.

### Genotyping for 14bp indel

**Fig 1:**
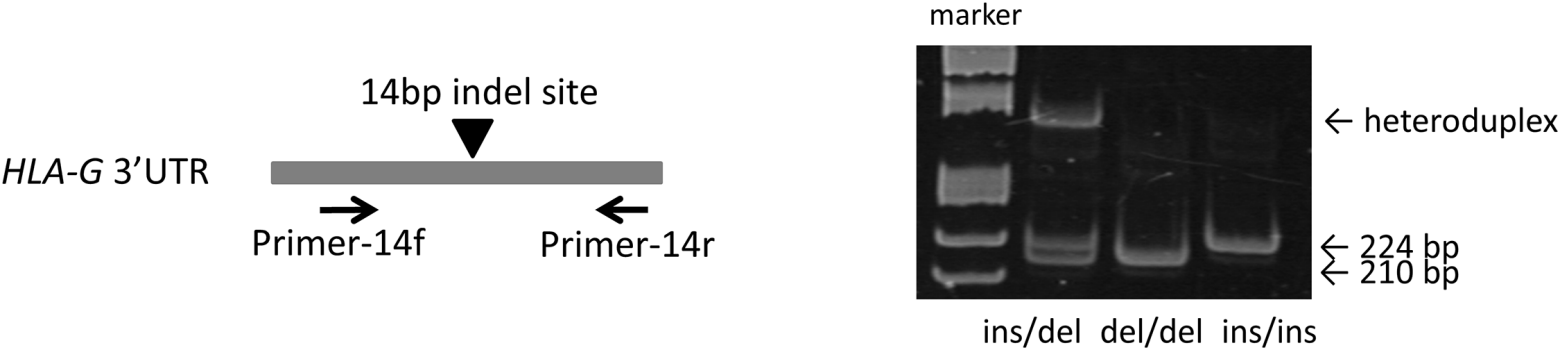
Genotyping for *HLA-G* 14 indel polymorphism. (Left) Primers were placed to encompass the 14bp indel site between primers. (Right) Polyacrylamide gel electrophoresis of the PCR products. The upper band in the ins/del lane shows the heteroduplex product formed by the ins and del allele products, which takes altered conformation and shows retardation in electrophoretic mobility. Ins, insertion; del, non-insertion.

Genotype of *HLA-G* 14bp indel was determined by multiplex PCR as previously described [13]. The sequence of the primers and probes used in this study are shown in S1 Table. AmpliTaq Gold^®^ 360 Master Mix (Applied Biosystems) 5 μL was used in 10 μL reaction mixture containing 0.1 μM of each primer. The PCR conditions consisted of initial denaturation at 95°C for 10 min, followed by 40 cycles of denaturation at 95°C for 30 s, annealing at 64°C for 60 s, and extension at 72°C for 30 s. The PCR products were detected by 7.5% acrylamide gel electrophoresis and staining using SYBR^®^ Gold nucleic acid gel stain (Life technologies) (Fig 1).

### Genotyping for *HLA-G* rs1063320

TaqMan SNP genotyping assay (ABI 7300, Applied Biosystems) was used to determine the genotype of rs1063320. Custom-designed TaqMan probes were purchased (S1 Table). For PCR, DNA samples (10–50 ng/μL) were added to the reaction mixture containing TaqMan^®^ Genotyping Master Mix (Applied Biosystems) and TaqMan probes. The PCR conditions consisted of initial denaturation at 95°C for 10 min, followed by 40 cycles of denaturation at 95°C for 15 s, annealing at 60°C for 60 s.

### Expression quantitative trait loci (eQTL) analysis

Association between *HLA-G* 14bp indel genotype and mRNA level of *HLA-G* was examined as follows. Genotypes of the HapMap JPT subjects for the 14bp indel were obtained from the 1000 Genomes project database (http://browser.1000genomes.org/index.html). The mRNA expression levels of *HLA-G* in the B lymphoblastoid cell lines from the same subjects were retrieved from GeneVar database (http://www.sanger.ac.uk/science/programmes) [31]. The probe GI_24797072 was placed within the 3’UTR and detect all isoforms of *HLA-G*. The difference in the mRNA levels between the genotypes was examined using regression analysis under the recessive model of 14bp ins allele.

### Statistical analysis

Association analysis was performed by logistic regression analysis under the additive model using R software (https://journal.r-project.org). First, “all SLE” group and each clinical subset were compared with healthy controls (case-control analysis). Next, the subsets which showed significant association in the case-control analysis were compared with the patients negative for the clinical characteristics (case-case analysis). P values for the case-control analysis and case-case analysis of overall SLE as well as each clinical subset at the two polymorphic sites (Tables 2–5, altogether 15 comparisons) were adjusted for false discovery rate (FDR) using the graphically sharpened method [32, 33]. FDR-adjusted P (P_FDR_) <0.05 was considered significant. Odds ratio (OR) was calculated for 14bp ins allele and rs1063320 C allele. Power calculation using Quanto program (http://biostats.usc.edu/Quanto.html) was conducted based on logistic regression analysis (S2 Table).

**Table 2:** Case-control analysis of 14bp indel polymorphism with SLE and its clinical subsets.

	n	genotype (%)			allele (%)	additive model			
		ins/ins	ins/del	del/del	Ins	P	P_FDR_	OR	(95%CI)
all SLE	843	56 (6.6)	351(41.6)	436(51.7)	463(27.5)	0.12		1.13	(0.97–1.33)
age of onset <20	144	17(11.8)	61(42.4)	66(45.8)	95(33.0)	0.0067	0.039	1.44	(1.10–1.87)
nephropathy (+)	397	25(6.3)	169(42.6)	203(51.1)	219(27.6)	0.19		1.14	(0.94–1.38)
anti-dsDNA Ab (+)	654	50(7.6)	264(40.4)	340(52.0)	364(27.8)	0.096		1.15	(0.98–1.36)
anti-Sm Ab (+)	252	17(6.7)	105(41.7)	130(51.6)	139(27.6)	0.26		1.14	(0.91–1.42)
anti-RNP Ab (+)	254	18(7.1)	97(38.2)	139(54.7)	133(26.2)	0.61		1.06	(0.85–1.32)
HC	777	58(7.5)	273(35.1)	446(57.4)	389(25.0)	ref			

Logistic regression analysis was used to test the association of 14bp indel under the additive model. Ins: insertion, del: non-insertion, OR: odds ratio, CI: confidence interval, ref: referent, Ab: antibody, HC: healthy controls.

**Table 3:** Case-case analysis on the association of 14bp ins polymorphism with early age of onset of SLE.

	n	genotype (%)			allele (%)	additive model			
		ins/ins	ins/del	del/del	ins	P	P_FDR_	OR	(95%CI)
age of onset <20	144	17(11.8)	61(42.4)	66(45.8)	95 (33.0)	0.033	0.0495	1.36	(1.02–1.80)
age of onset ≥20	632	38 (6.0)	263 (41.6)	331(52.4)	339 (26.8)	ref			

Logistic regression analysis was performed to compare the 14bp indel genotypes between SLE patients with the age of onset <20 and ≥20 years. OR: odds ratio, CI: confidence interval.

**Table 4:** Case-control analysis of rs1063320 with SLE and its clinical subsets.

	n	genotype (%)			allele (%)	additive model			
		G/G	G/C	C/C	C	P	P_FDR_	OR	(95%CI)
all SLE	843	408(48.4)	349(41.4)	86(10.2)	521(30.9)	0.096		1.14	(0.98–1.32)
age of onset <20	144	76(52.8)	58(40.3)	10(6.9)	78(27.1)	0.69		0.94	(0.71–1.25)
nephropathy (+)	397	189(47.6)	169(42.6)	39(9.8)	247(31.1)	0.14		1.15	(0.95–1.39)
anti-dsDNA Ab (+)	654	328(50.2)	257(39.3)	69(10.6)	395(30.2)	0.25		1.10	(0.94–1.29)
anti-Sm Ab (+)	252	116(46.0)	102(40.5)	34(13.5)	170(33.7)	0.020	0.040	1.29	(1.04–1.60)
anti-RNP Ab (+)	254	114(44.9)	107(42.1)	33(13.0)	173(34.1)	0.013	0.039	1.31	(1.06–1.62)
HC	778	399(51.3)	319(41.0)	60(7.7)	439(28.2)	ref			

OR was calculated for the C allele. HC: healthy controls, OR: odds ratio, CI: confidence interval, ref: referent, Ab: antibody.

**Table 5:** Case-case analysis on the association of rs1063320 with anti-Sm and anti-RNP antibody-positive SLE and antibody-negative SLE.

	n	genotype (%)			allele (%)	additive model		
		G/G	G/C	C/C	C	P	OR	(95%CI)
anti-Sm Ab (+) SLE	252	116(46.0)	102(40.5)	34(13.5)	170(33.7)	0.082	1.22	(0.97–1.52)
anti-Sm Ab (-) SLE	523	265(50.7)	210(40.2)	48(9.2)	206(29.3)	ref		
anti-RNP Ab (+) SLE	254	114(44.9)	107(42.1)	33(13.0)	173(34.1)	0.15	1.19	(0.94–1.51)
anti-RNP Ab (-) SLE	374	185(49.5)	153(40.9)	36(9.6)	225(30.1)	ref		

Logistic regression analysis was performed to compare the rs1063320 genotypes between SLE patients positive (+) and negative (-) for anti-Sm and anti-RNP antibodies (Ab). OR: odds ratio, CI: confidence interval, ref, referent.

Association of the 14bp indel genotype with the age of onset of SLE was examined using regression analysis.

Conditional logistic regression analysis was used to evaluate contribution of each polymorphism among the polymorphisms in potential LD.

Calculation for LD coefficient *D’* and *r*^*2*^, estimation of haplotype frequencies, and permutation test were performed using HaploView software [34]. Permutation for 100,000 times was conducted for haplotype analysis.

## Results

### Association study of *HLA-G* 14bp indel

Genotype frequency of 14bp indel in the healthy controls was not significantly departed from Hardy-Weinberg equilibrium (P = 0.075). Significant association was not observed in overall SLE. When the clinical subsets of SLE were analyzed, significant association of 14bp ins allele was observed in early-onset (age<20) SLE both in case-control analysis (P = 0.0067, P_FDR_ = 0.039, OR 1.44, 95%CI 1.10–1.87) (Table 2) and in case-case analysis (P = 0.033, P_FDR_ = 0.0495, OR 1.36, 95%CI 1.02–1.80) (Table 3). Association of 14bp indel was also observed with actual age of onset of each patient by regression analysis under the recessive model of the ins allele (β = -4.54, 95%CI -8.55 –-0.52, P = 0.027).

### Association study of rs1063320 with SLE

Genotype frequency in the healthy controls were not significantly departed from Hardy-Weinberg equilibrium (P = 0.73). The C allele of the SNP rs1063320 showed significant increase in the SLE patients positive for anti-Sm antibody (P = 0.020, P_FDR_ = 0.040, OR 1.29, 95%CI 1.04–1.60) and in those positive for anti-RNP antibody P = 0.013, P_FDR_ = 0.039, OR 1.31, 95%CI 1.06–1.62) when compared with healthy controls (Table 4).

In the case-case analysis between the antibody positive and negative patients, the association did not reach statistical significance, although the tendency for stronger association was observed in the antibody positive subsets (Table 5).

### Analysis of independent contribution of *HLA-G* from *HLA-DRB1*

Because DRB1*15:*01* and DRB1*13:*02* are associated with risk and protection of SLE, respectively, we performed conditional logistic regression analysis to investigate the independent contribution of *HLA-G* from *DRB1* alleles on 827 patients and 576 healthy controls in whom we already genotyped *DRB1* alleles [8]. *DRB1* allele frequencies of the patients and healthy controls as well as the results of association analysis are shown in S3 Table.

**Fig 2:**
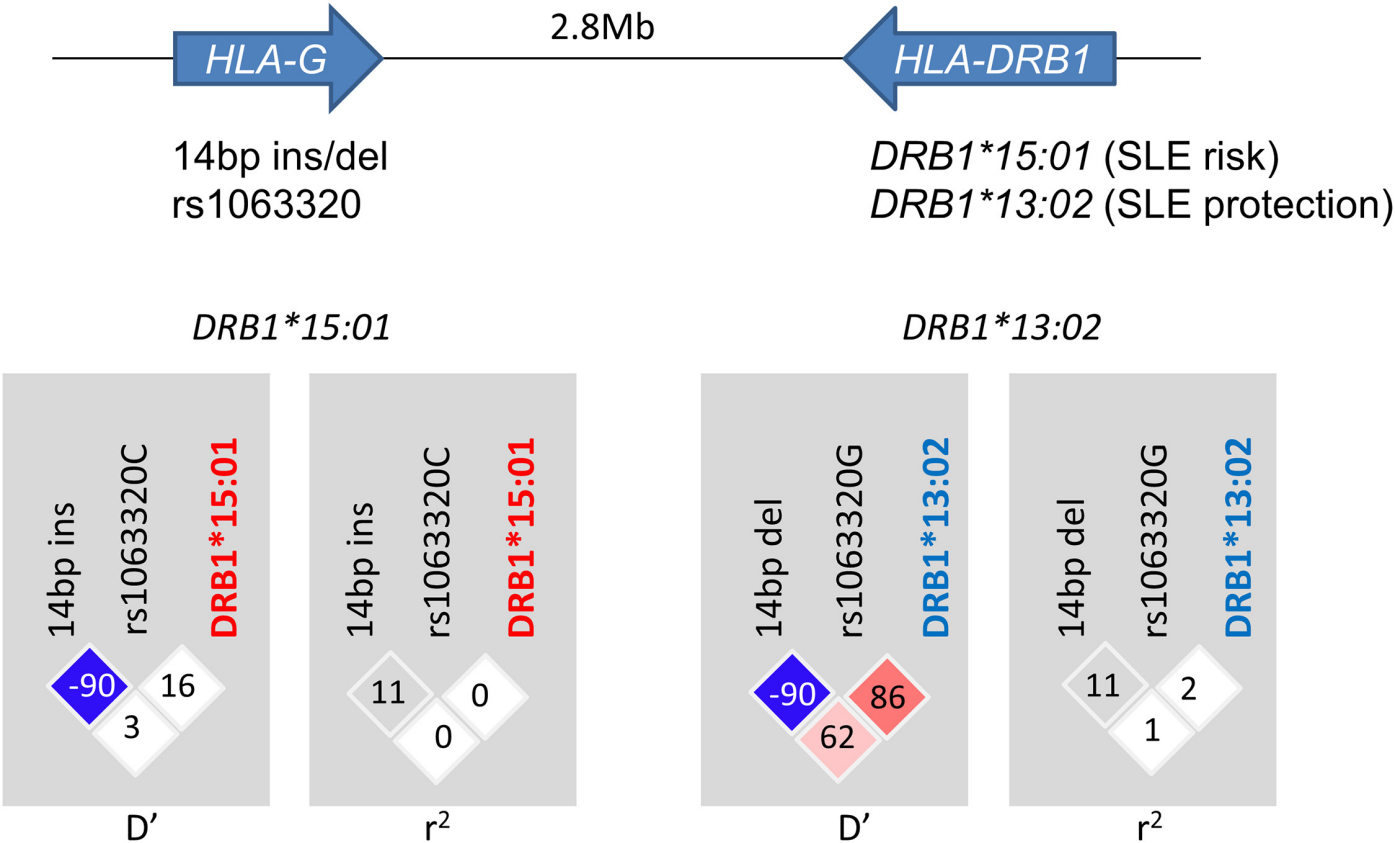
Linkage disequilibrium parameters between *HLA-G* 14bp indel, rs1063320 and DRB1*15:*01* or DRB1*13:*02*. LD was detected between DRB1*13:*02* and *HLA-G* 14bp del and, more strongly, rs1063320 G. Only weak LD was observed between DRB1*15:*01* and *HLA-G* alleles. Negative LD was observed between *HLA-G* 14 bp ins and rs1063320 C (shown in blue matrix in the LD plot).

LD plots between DRB1*15:*01*, DRB1*13:*02* and *HLA-G* 3’UTR polymorphisms in our healthy controls are shown in Fig 2. LD was detected between SLE-protective DRB1*13:*02* and *HLA-G* 14bp del (*D’* = 0.62, *r*^*2*^ = 0.01) and, more strongly, rs1063320 G (*D’* = 0.86, *r*^*2*^ = 0.02). On the other hand, only weak LD was observed between SLE-susceptible DRB1*15:*01* and *HLA-G* 14 bp ins (*D’* = 0.03, *r*^*2*^ = 0.00) and rs1063320 C (*D’* = 0.16, *r*^*2*^ = 0.00). Negative LD was observed between *HLA-G* 14 bp ins and rs1063320 C (*D’* = -0.90, r^2^ = 0.11).

The association between *HLA-G* 14bp ins and early-onset SLE remained significant when conditioned on DRB1*15:*01* and DRB1*13:*02*, both in the case-control analysis and case-case analysis (Table 6). On the other hand, the association of rs1063320 with anti-Sm antibody and anti-RNP antibody positive SLE lost significance when conditioned on DRB1*13:*02* (Table 6).

**Table 6:** Conditional logistic regression analysis to evaluate contribution of *HLA-G* genotypes when conditioned on HLA-DRB1*15:*01* and DRB1*13:*02*.

	Conditioned on:								
	none			DRB1*15:*01*			DRB1*13:*02*		
	P	OR	(95%CI)	P	OR	(95%CI)	P	OR	(95%CI)
14bp ins									
age of onset <20 vs HC	0.0067	1.44	(1.10–1.87)	0.032	1.36	(1.02–1.80)	0.044	1.33	(1.01–1.76)
age of onset <20 vs ≥20	0.033	1.36	(1.02–1.80)	0.046	1.34	(1.00–1.77)	0.049	1.33	(1.00–1.77)
rs1063320 C									
anti-Sm Ab (+) vs HC	0.020	1.29	(1.04–1.60)	0.062	1.24	(0.99–1.56)	0.16	1.18	(0.94–1.48)
anti-RNP Ab (+) vs HC	0.013	1.31	(1.06–1.62)	0.072	1.23	(0.98–1.55)	0.15	1.18	(0.94–1.49)

Conditional logistic regression analysis demonstrated independent contribution of *HLA-G* 14bp ins on the age of onset of SLE, but the association of rs1063320 C on the production of anti-Sm and anti-RNP antibodies was dependent on LD with *DRB1* alleles. OR was calculated for the ins or C allele. OR: odds ratio, CI: confidence interval, ref: referent, Ab: antibody, HC: healthy controls.

In contrast, the association of DRB1*15:*01* and DRB1*13:*02* was unaffected when conditioned on *HLA-G* alleles (S4 Table).

These results implied that *HLA-G* 14bp ins allele has an independent contribution for early-onset SLE, while the association of rs1063320 C with anti-Sm and anti-RNP production is dependent on LD between the protective DRB1*13:*02* and rs1063320 G allele.

#### Association study of the haplotypes formed by *HLA-G* and *DRB1*

We next estimated the haplotype frequencies formed by HLA-G alleles and DRB1*13:02 (Table 7). Four haplotypes with haplotype frequencies >1% were estimated using HaploView [34]. DRB1*13:*02* was present only on the *HLA-G* 14bp del-rs1063320 G haplotype. Significant protection against overall SLE was observed for 14bp del-rs1063320 G haplotype carrying DRB1*13:*02*, but not for the same *HLA-G* haplotype carrying other *DRB1* alleles, suggesting that the protective effect against overall SLE is mediated by DRB1*13:*02* rather than *HLA-G*. Similarly, protection against anti-Sm or anti-RNP antibody positive SLE was observed only for the haplotype carrying DRB1*13:*02*.

**Table 7:** Estimation of haplotype frequencies constituted by *HLA-G* 14bp indel, rs1063320 and DRB1*13:*02* and association analysis.

haplotype			estimated haplotype frequency (%)					P_permutation_ vs HC			
14bp	rs1063320	*DRB1*	all SLE	early-onset	anti-Sm Ab (+)	anti-RNP Ab (+)	HC	all SLE	early-onset	anti-Sm Ab (+)	anti-RNP Ab (+)
del	G	other	38.6	37.4	39.9	40.1	39.1	0.99	0.96	1.0	1.0
del	C	other	30.5	26.8	27.3	26.3	27.3	0.17	1.0	1.0	0.98
ins	G	other	26.7	32.0	28.0	27.7	24.3	0.38	0.020	0.12	0.17
del	G	*13*:*02*	3.3	2.9	3.8	4.3	7.6	1.0 x 10^−5^	0.011	0.0089	0.038

Haplotype estimation and association analysis was conducted by permutation test using HaploView. 100,000 times permutation was conducted. The haplotypes with frequencies more than 1% are shown. Independent effect of *HLA-G* 14bp ins-G haplotype from DRB1*13:*02* was supported by this analysis. Early-onset: SLE with the age of onset <20; Ab: antibody, HC: healthy controls.

In contrast, 14bp ins was present essentially on the haplotypes not carrying DRB1*13:*02*. Among such haplotypes, only those carrying 14bp ins demonstrated significant increase in early-onset SLE, suggesting that 14bp ins itself is responsible for, or tags the functional variant associated with, early-onset of SLE.

### eQTL analysis of *HLA-G* 14bp indel.

**Fig 3:**
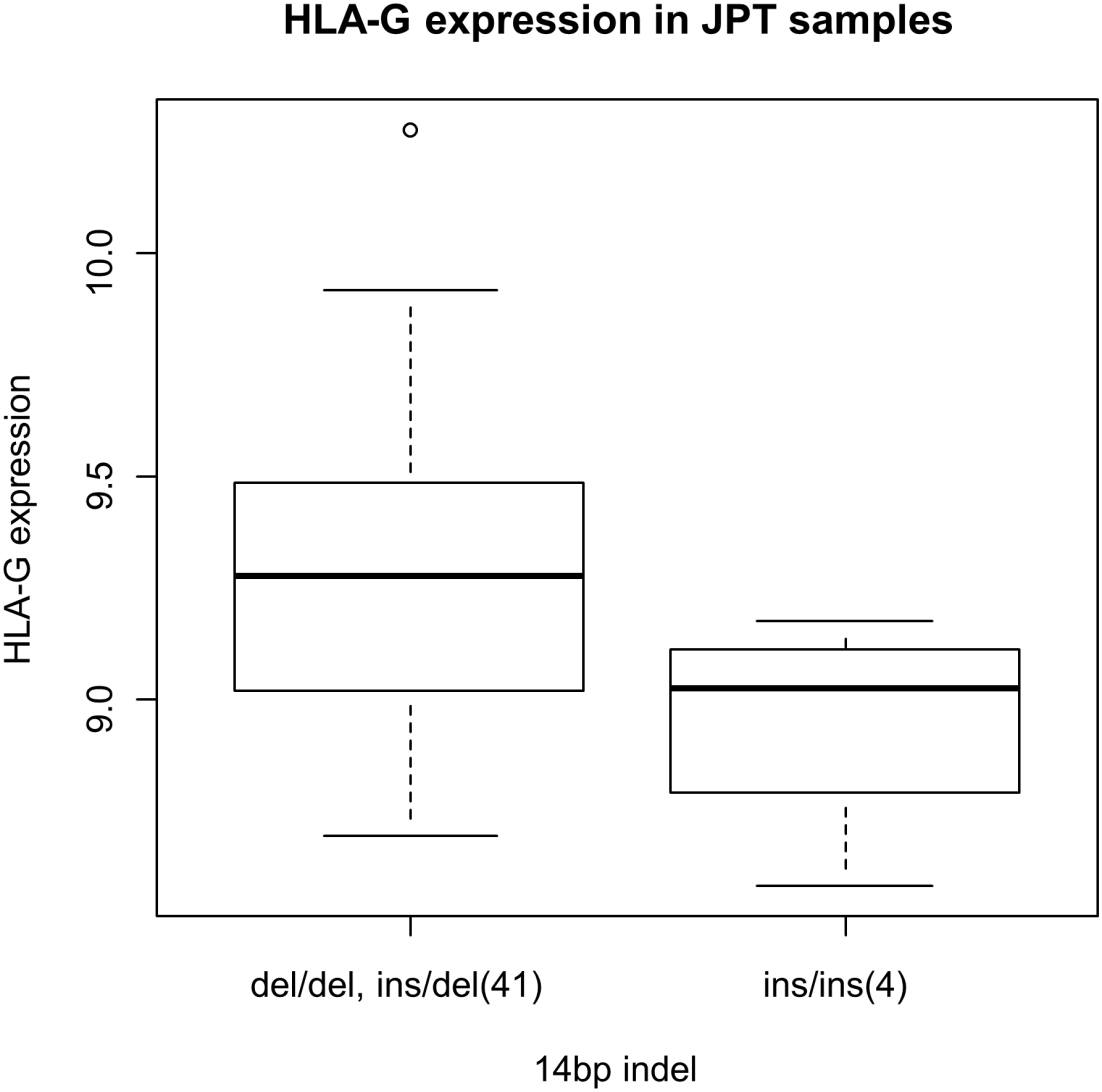
Decreased *HLA-G* mRNA level in 14bp ins/ins genotype. *HLA-G* mRNA levels in the B lymphoblastoid cell lines were compared between HapMap JPT individuals with 14bp del/del or ins/del genotypes and ins/ins genotype using regression analysis. Significant decrease was detected in individuals with ins/ins genotype β = -0.338, 95%CI -0.672 –-0.005, P = 0.047). The number of the subjects in each group is shown in the parentheses.

To elucidate the effect of *HLA-G* 14bp indel on the mRNA expression, eQTL analysis was performed using the genotype data of the HapMap JPT samples and *HLA-G* mRNA levels in the B lymphoblastoid cell lines derived from the same subjects. As shown in Fig 3, *HLA-G* mRNA level was significantly lower in individuals with 14bp ins/ins genotype (β = -0.338, 95%CI -0.672 –-0.005, P = 0.047).

## Discussion

In the present study, we conducted an association study of two *HLA-G* 3’UTR polymorphisms, previously suggested to have functional significance, with SLE for the first time in a Japanese population. Our sample size was probably the largest among the *HLA-G* association studies with SLE. In addition, we took advantage of our previous study in which we genotyped SLE patients and healthy controls for the *HLA-DRB1* allele at the high resolution (“four-digit”) level [8], and tested for the independent association of *HLA-G*. We detected association of *HLA-G* 3’UTR 14bp ins (rs371194629) with SLE, and rs1063320 C with anti-RNP and anti-Sm antibody positive SLE. SLE-protective HLA-DRB1*13:*02* allele was found to be in LD with *HLA-G* 14 bp del and rs1063320 G (Fig 2). Thus, the obvious question is whether the increase of *HLA-G* 14 bp and rs1063320 C may be secondarily caused by the decrease of DRB1*13:*02*. In the case of *HLA-G* 14bp ins, three findings support the “independent” contribution of *HLA-G* 14 bp.

First, *HLA-G* 14bp ins was significantly associated with SLE with the age of onset <20 even after conditioning on DRB1*13:*02* status (Table 6). Next, increase of *HLA-G* 14 bp ins was observed not only when compared with healthy controls, but also in the case-case analysis which compared SLE patients with the age of onset <20 and ≥ 20 years, while DRB1*13:*02* did not show significant association in the case-case analysis (S4 Table). Finally, in the haplotype analysis, only the haplotype with *HLA-G* 14 bp ins was increased among the three haplotypes which do not carry DRB1*13:*02* (Table 7).

In contrast, association of rs1063320 C with anti-RNP antibody or anti-Sm antibody positive SLE lost significance when conditioned on DRB1*13:*02* (Table 6). Thus, we concluded that the association of this SNP was caused by negative LD with DRB1*13:*02*.

Association of *HLA-G* 3’UTR polymorphisms with SLE has been reported with inconsistent results [6, 15–21]. Although *HLA-class II* and *HLA-G* genes are located at the opposite ends of the *MHC* region and are 2.8 Mb distant from each other, several studies reported LD between them [12, 26–28, 35]. With respect to *HLA-G* association studies with SLE, only one has considered LD with *HLA-class II* haplotypes [6]. It is likely that difference in the LD between *HLA-class II* and *HLA-G* might be related to the inconsistencies among the results of the previous association studies.

DRB1*13:*02* is carried by a long-range extended haplotype A*33:03-C*14:03-B*44:03-DRB1*13:02-DQB1*06:04-DPB1*04:*01*, which shows evidence for positive selection in the Japanese population [29]. This haplotype was reported to be present in Korean populations, but may not be present in other Asian populations [29]. Among our subjects, 172 healthy controls had been genotyped for *HLA-A*. LD between A*33:*03* and *HLA-G* rs1063320 G (*D’* = 1, *r*^*2*^ = 0.051) and also between A*33:*03* and *HLA-G*14bp del (*D’* = 1, *r*^*2*^ = 0.043) were detected. Similarly, all other *HLA* alleles on the extended haplotype are expected to be in LD. Thus, to untangle contribution of each allele in LD, allele typing of all the classical *HLA-class I* and *class II* alleles should eventually be carried out in all the patients and controls, which is beyond the scope of this study. Thus, at this point, it would be reasonable to conclude that *HLA-G* 14bp ins represents a genetic contribution on early-onset SLE independent of *DRB1*.

In our study, the association of 14bp ins with early-onset SLE remained significant after conditioning on DRB1*13:*02* and DRB1*15:*01* alleles. Although 14bp ins has been shown to be in strong LD with DRB1*03:*01* [28, 35], the major risk allele for SLE in the Caucasian populations, DRB1*03:*01* is very rare in the Japanese population (allele frequency 0.1% [29]), and cannot explain the association of 14bp ins. Thus, the Japanese population provided a unique opportunity to distinguish the association of *HLA-G* 14bp and DRB1*03:*01*. On the other hand, the association of DRB1*15:*01* remained strong after conditioning on 14bp ins. Thus, it is considered that DRB1*15:*01* and *HLA-G* 14bp ins, or other alleles in strong LD with them, have independent genetic contribution for susceptibility to SLE.

*HLA-G* 14bp ins was not found to be associated with specific clinical symptoms, but association was mainly observed in the patients with early age of onset <20 years. The association was also significant when compared with those with the age of onset ≥20 years in case-case analysis (Table 3), and regression analysis among the patients. This is probably because the SLE patients with younger onset are associated with higher genetic risk [36] and susceptibility alleles were more sensitively detectable in such patients.

Although statistically not significant, the genotype distribution in the healthy controls showed slight tendency toward deviation from Hardy-Weinberg equilibrium (P = 0.075). Although we could not find other association studies of 14bp indel in the Japanese population in the literature, we were able to retrieve small population data from the 1000 Genomes Browser (http://www.ncbi.nlm.nih.gov/variation/tools/1000genomes/): ins/ins 6 (5.8%), ins/del 40 (38.5%), del/del 58 (55.8%). When this control set is used instead of our healthy subjects, the association of early-onset SLE barely missed significance level (P = 0.059) due to the small sample size of the controls; however, the OR was 1.47 (95%CI 0.99–2.19), and was slightly higher than the OR observed with our controls (1.44, Table 2). Thus, we think that the detected association was not merely caused by the slight tendency toward deviation from Hardy-Weinberg equilibrium of the healthy control genotype frequencies.

Although previous reports demonstrated association of *HLA-G* 14bp indel with SLE and various autoimmune diseases including celiac disease, pemphigus vulgaris and multiple sclerosis, the genetic association as well as its functional consequences are not yet established [10, 11]. Most studies demonstrated decreased sHLA-G level in individuals with the14bp ins/ins genotype [15, 16], although conflicting results are reported [14]. By combining the sequencing data of the HapMap subjects from the 1000 genomes project database and the expression data from the GeneVar database, we confirmed the low *HLA-G* mRNA level in individuals with 14bp ins/ins genotype (Fig 3).

*HLA-G* has seven mRNA isoforms generated by alternative splicing, among which three code for sHLA-G. sHLA-G is present as monomers or dimers, and each can be present in β_2_-microglobulin associated and unassociated forms, potentially having different conformation and affinity with various monoclonal antibodies. This makes it difficult to accurately quantitate both membrane-bound HLA-G and sHLA-G protein. Genotyping of genomic polymorphisms may possibly offer more stable and technically undemanding biomarker as compared with detection of membrane-bound or sHLA-G at the protein level.

HLA-G has been shown to be involved in inhibition of NK cells, neutrophils, dendritic cells, T cells and B cells via *LILRB1* and *LILRB2* (reviewed in [11]). Thus, lower level of sHLA-G may account for the excessive activation observed in multiple immune cell types in SLE. Of interest, deletion polymorphism encompassing the large part of *LILRA3*, a soluble receptor which possibly antagonizes binding of sHLA-G and LILRB1/LILRB2, has been associated with a variety of immune system disorders [37]. A recent study reported association of *LILRA3* non-deletion allele encoding functional LILRA3 molecule with susceptibility to SLE [38]. This observation is in line with the idea that decreased inhibitory signal from sHLA-G may be associated with the development of SLE. Genetic interaction between *LILR* and *HLA-G* genotypes involved in the occurrence of autoimmune diseases will be an intriguing subject of study in the future.

Although association between *HLA-G* 14bp ins with SLE has previously been reported, conditional analysis for the independent association as well as the association with early-onset SLE need to be replicated in future studies.

In conclusion, this study demonstrated association of *HLA-G* 14bp ins allele with early-onset SLE in the Japanese population. This association was independent from the association of DRB1*15:*01* with the risk and DRB1*13:*02* with the protection of SLE. Further studies will be necessary to establish the extended *MHC* haplotype dependent and independent roles of *HLA-G* polymorphisms.

## Supporting Information

S1 Table Sequence of the primers and probes used in this study. (DOCX)

S2 Table Power calculation under the additive model based on logistic regression. (DOCX)

S3 Table Frequencies and associations of *HLA-DRB1* alleles in all SLE patients and healthy controls. (DOCX)

S4 Table Conditional logistic regression analysis to evaluate contribution of HLA-DRB1*15:*01* and DRB1*13:*02* genotypes when conditioned on *HLA-G* 14bp indel and rs1063320. (DOCX)
