# Supplementary material for: Association of HLA-G 3’ Untranslated Region Polymorphisms with Systemic Lupus Erythematosus in a Japanese Population: A Case-Control Association Study
Source: PLoS One. 2016 Jun 22;11(6):e0158065. doi: 10.1371/journal.pone.0158065 (PMC4917238; doi:10.1371/journal.pone.0158065)
Supplement: S1 Table — (DOCX) [file pone.0158065.s001.docx]

**S1 Table. Sequence of the primers and probes used in this study.**

| Polymorphisms | Primer/Probe | Sequence (5'-3') | Reference |
| --- | --- | --- | --- |
| 14bp indel | Primer-14f | GTGATGGGCTGTTTAAAGTGTCACC | [13] |
|  | Primer-14r | GGAAGGAATGCAGTTCAGCATGA | [13] |
| rs1063320 | Forward primer | ATCAATCTCTCTTGGAAATAATTTGAAAAA |  |
|  | Reverse primer | CCTTCCCCAATCACCTTTCCT |  |
|  | Reporter probe 1 | TTATAGCTCAGTGGACCACA |  |
|  | Reporter probe 2 | TAGCTCAGTGCACCACA |  |

The primers and probes for rs1063320 were custom-designed (Applied Biosystems).
