# Supplementary material for: Association of HLA-G 3’ Untranslated Region Polymorphisms with Systemic Lupus Erythematosus in a Japanese Population: A Case-Control Association Study
Source: PLoS One. 2016 Jun 22;11(6):e0158065. doi: 10.1371/journal.pone.0158065 (PMC4917238; doi:10.1371/journal.pone.0158065)
Supplement: S2 Table — (DOCX) [file pone.0158065.s002.docx]

**S2 Table. Power calculation under the additive model based on logistic regression.**

|  | 14bp indel (Po = 0.25) | | rs1063320 (Po =0.282) | |
| --- | --- | --- | --- | --- |
|  | n | OR^*^ | n | OR^†^ |
| all SLE | 843 | 1.25 | 843 | 1.24 |
| age of onset <20 | 144 | 1.49 | 144 | 1.47 |
| nephropathy (+) | 397 | 1.32 | 397 | 1.31 |
| anti-dsDNA Ab (+) | 654 | 1.27 | 654 | 1.26 |
| anti-Sm Ab (+) | 252 | 1.38 | 252 | 1.37 |
| anti-RNP Ab (+) | 254 | 1.38 | 254 | 1.37 |
| HC | 777 |  | 778 |  |

The odds ratios (OR) when the detection power larger than 80% are expected with significant level at 0.05 are shown. Ab: antibody, HC: healthy controls, Po: risk allele frequencies in the healthy controls, ^*^OR for the insertion allele, ^†^ OR for the C allele.
