# Supplementary material for: Association of HLA-G 3’ Untranslated Region Polymorphisms with Systemic Lupus Erythematosus in a Japanese Population: A Case-Control Association Study
Source: PLoS One. 2016 Jun 22;11(6):e0158065. doi: 10.1371/journal.pone.0158065 (PMC4917238; doi:10.1371/journal.pone.0158065)
Supplement: S3 Table — (DOCX) [file pone.0158065.s003.docx]

**S3 Table. Frequencies and associations of *HLA-DRB1* alleles in all SLE patients and healthy controls.**

| *DRB1* | all SLE(%) (n=827) | HC (%)  (n=576) | P | P_FDR_ | OR | (95%CI) |
| --- | --- | --- | --- | --- | --- | --- |
| **01:01* | 69 (4.2) | 66 (5.7) | 0.060 |  | 0.72 | (0.50–1.03) |
| **04:01* | 36 (2.2) | 17 (1.5) | 0.21 |  | 1.49 | (0.81–2.83) |
| **04:03* | 30 (1.8) | 27 (2.3) | 0.34 |  | 0.77 | (0.44–1.35) |
| **04:05* | 182 (11.0) | 143 (12.4) | 0.26 |  | 0.87 | (0.69–1.11) |
| **04:06* | 25 (1.5) | 37 (3.2) | 0.0037 | 0.010 | 0.46 | (0.27–0.79) |
| **04:10* | 24 (1.5) | 16 (1.4) | 1.00 |  | 1.05 | (0.53–2.12) |
| **08:02* | 96 (5.8) | 34 (3.0) | 0.00035 | 0.0016 | 2.03 | (1.35–3.11) |
| **08:03* | 160 (9.7) | 82 (7.1) | 0.020 | 0.040 | 1.40 | (1.05–1.87) |
| **09:01* | 269 (16.3) | 176 (15.3) | 0.50 |  | 1.08 | (0.87–1.33) |
| **11:01* | 25 (1.5) | 21 (1.8) | 0.55 |  | 0.83 | (0.44–1.56) |
| **12:01* | 78 (4.7) | 46 (4.0) | 0.40 |  | 1.19 | (0.81–1.77) |
| **12:02* | 27 (1.6) | 22 (1.9) | 0.66 |  | 0.85 | (0.46–1.58) |
| **13:02* | 67 (4.1) | 102 (8.9) | 2.7 x 10^-7^ | 1.9 x 10^-6^ | 0.43 | (0.31–0.60) |
| **14:03* | 15 (0.9) | 27 (2.3) | 0.0025 | 0.0088 | 0.38 | (0.19–0.74) |
| **14:05* | 25 (1.5) | 34 (3.0) | 0.011 | 0.026 | 0.50 | (0.29–0.88) |
| **14:06* | 12 (0.7) | 12 (1.0) | 0.41 |  | 0.69 | (0.28–1.70) |
| **14:54* | 37 (2.2) | 32 (2.8) | 0.39 |  | 0.80 | (0.48–1.34) |
| **15:01* | 231 (14.0) | 76 (6.6) | 3.8 x 10^-10^ | 4.2 x 10^-9^ | 2.30 | (1.74–3.06) |
| **15:02* | 159 (9.6) | 128 (11.1) | 0.21 |  | 0.85 | (0.66–1.10) |

*HLA-DRB1* allele frequencies of all SLE patients and healthy controls. P values were calculated by Fisher’s exact test. FDR-based adjustment for multiple testing of 19 *DRB1* alleles was performed using the graphically sharpened method [32,33]. Allele frequencies of healthy controls more than 1% are shown. HC: healthy controls, OR: odds ratio, P_FDR_: P value adjusted for FDR, CI: confidence interval, NS: not significant.
