# Supplementary material for: Association of HLA-G 3’ Untranslated Region Polymorphisms with Systemic Lupus Erythematosus in a Japanese Population: A Case-Control Association Study
Source: PLoS One. 2016 Jun 22;11(6):e0158065. doi: 10.1371/journal.pone.0158065 (PMC4917238; doi:10.1371/journal.pone.0158065)
Supplement: S4 Table — (DOCX) [file pone.0158065.s004.docx]

**S4 Table. Conditional logistic regression analysis to evaluate contribution of *HLA-DRB1*15:01* and *DRB1*13:02* genotypes when conditioned on *HLA-G* 14bp indel and rs1063320.**

|  | Conditioned on: | | | | | | | | |
| --- | --- | --- | --- | --- | --- | --- | --- | --- | --- |
|  | none | | | 14bp indel | | | rs1063320 | | |
|  | P | OR | (95%CI) | P | OR | (95%CI) | P | OR | (95%CI) |
| all SLE vs HC |  |  |  |  |  |  |  |  |  |
| *DRB1*15:01* | 6.2 x 10^-9^ | 2.22 | (1.71–2.92) | 6.9 x 10^-9^ | 2.21 | (1.70–2.91) | 1.3 x 10^-8^ | 2.19 | (1.68–2.89) |
| *DRB1*13:02* | 2.6 x 10^-7^ | 0.43 | (0.31–0.59) | 3.5 x 10^-7^ | 0.43 | (0.31–0.59) | 7.0 x 10^-7^ | 0.44 | (0.31–0.60) |
| age of onset <20 vs HC |  |  |  |  |  |  |  |  |  |
| *DRB1*15:01* | 2.9 x 10^-5^ | 2.32 | (1.56–3.45) | 5.2 x 10^-5^ | 2.27 | (1.52–3.37) | 2.6 x 10^-5^ | 2.34 | (1.57–3.48) |
| *DRB1*13:02* | 0.0034 | 0.37 | (0.18–0.68) | 0.0059 | 0.39 | (1.01–1.76) | 7.0 x 10 ^-7^ | 0.44 | (0.31–0.60)) |
| age of onset <20 vs ≥20 |  |  |  |  |  |  |  |  |  |
| *DRB1*15:01* | 0.63 | 1.09 | (0.75–1.56) | 0.63 | 1.09 | (0.75–1.56) | 0.50 | 1.13 | (0.78–1.62) |
| *DRB1*13:02* | 0.45 | 0.77 | (0.36–1.46) | 0.48 | 0.78 | (0.37–1.49) | 0.34 | 0.95 | (0.86–1.05)) |
| anti-Sm Ab(+) vs HC |  |  |  |  |  |  |  |  |  |
| *DRB1*15:01* | 1.3 x 10^-4^ | 1.97 | (1.39–2.79) | 1.3 x 10^-4^ | 1.96 | (1.39–2.78) | 2.8 x 10^-4^ | 1.91 | (1.35–2.70) |
| *DRB1*13:02* | 7.3 x 10^-5^ | 0.33 | (0.19–0.56) | 4.1 x 10^-5^ | 0.83 | (0.76–0.91) | 2.2 x 10^-4^ | 0.35 | (0.20–0.60) |
| anti-RNP Ab(+) vs HC |  |  |  |  |  |  |  |  |  |
| *DRB1*15:01* | 5.0 x 10^-6^ | 2.18 | (1.56–3.05) | 5.2 x 10^-6^ | 2.18 | (1.56–3.05) | 1.7 x 10^-5^ | 2.10 | (1.50–2.94) |
| *DRB1*13:02* | 4.1 x 10^-6^ | 0.22 | (0.11–0.40) | 3.7 x 10^-6^ | 0.22 | (0.11–0.40) | 1.1 x 10^-5^ | 0.24 | (0.12–0.43) |

Association of *DRB1*15:01* with risk and *DRB1*13:02* with protection of SLE remained significant after conditioning on *HLA-G* 14bp indel and rs1063320. HC: healthy controls.
